# Relationship between Delta Rhythm, Seizure Occurrence and Allopregnanolone Hippocampal Levels in Epileptic Rats Exposed to the Rebound Effect

**DOI:** 10.3390/ph14020127

**Published:** 2021-02-06

**Authors:** Anna-Maria Costa, Chiara Lucchi, Asiye Malkoç, Cecilia Rustichelli, Giuseppe Biagini

**Affiliations:** 1 Laboratory of Experimental Epileptology, Department of Biomedical, Metabolic and Neural Sciences, University of Modena and Reggio Emilia, 41125 Modena, Italy; annamaria.costa@unimore.it (A.-M.C.); chiara.lucchi@unimore.it (C.L.); asiye.malkocc@gmail.com (A.M.); 2 Department of Life Sciences, University of Modena and Reggio Emilia, 41125 Modena, Italy; cecilia.rustichelli@unimore.it; 3 Center for Neuroscience and Neurotechnology, University of Modena and Reggio Emilia, 41125 Modena, Italy

**Keywords:** delta rhythm, epilepsy, kainic acid, levetiracetam, neurosteroids, postictal, rebound effect

## Abstract

Abrupt withdrawal from antiepileptic drugs is followed by increased occurrence of epileptic seizures, a phenomenon known as the “rebound effect”. By stopping treatment with levetiracetam (LEV 300 mg/kg/day, *n* = 15; vs. saline, *n* = 15), we investigated the rebound effect in adult male Sprague-Dawley rats. LEV was continuously administered using osmotic minipumps, 7 weeks after the intraperitoneal administration of kainic acid (15 mg/kg). The effects of LEV were determined by comparing time intervals, treatments, and interactions between these main factors. Seizures were evaluated by video-electrocorticographic recordings and power band spectrum analysis. Furthermore, we assessed endogenous neurosteroid levels by liquid chromatography-electrospray-tandem mass spectrometry. LEV significantly reduced the percentage of rats experiencing seizures, reduced the seizure duration, and altered cerebral levels of neurosteroids. In the first week of LEV discontinuation, seizures increased abruptly up to 700% (*p* = 0.002, Tukey’s test). The power of delta band in the seizure postictal component was related to the seizure occurrence after LEV withdrawal (*r*^2^ = 0.73, *p* < 0.001). Notably, allopregnanolone hippocampal levels were positively related to the seizure occurrence (*r*^2^ = 0.51, *p* = 0.02) and to the power of delta band (*r*^2^ = 0.67, *p* = 0.004). These findings suggest a role for the seizure postictal component in the rebound effect, which involves an imbalance of hippocampal neurosteroid levels.

## 1. Introduction

Epilepsy is a chronic neurological disorder affecting over 50 million people worldwide [1]. Approximately 65% of patients remain seizure-free after undergoing treatment with antiepileptic drugs (AEDs) as a single administration or in combined therapy [2]. However, the only possibility to overcome multidrug resistance in patients with refractory seizures is surgical resection of the epileptic foci. For instance, temporal lobectomy is the most effective treatment for refractory temporal lobe epilepsy [3], which is the most common form of medically intractable epilepsy in adults [4].

In epileptic patients who are candidates for surgical treatment, the rapid discontinuation of AEDs is required during the presurgical evaluation of video-electroencephalogram (v-EEG) monitoring [5,6,7,8]. However, this procedure can trigger the so-called “rebound effect”, which is characterized by transient seizure generalization or even prolonged increase in frequency of partial seizures [5]. Other adverse events observed during the presurgical evaluation of v-EEG monitoring could be the *status epilepticus* or sudden unexpected death in epilepsy [9,10]. These are dramatic events, but also uncontrolled seizures during the rebound effect could be damaging, because they were associated with development of brain hypoxia and neurodegeneration [11].

In general, the clinical effects of AED rapid discontinuation might depend on predicting factors of relapse, such as: (i) age, (ii) family history of epilepsy, (iii) etiology, (iv) EEG abnormalities, (v) number of seizures before discontinuation, vi) duration of seizure-free period on treatment, and vii) number of AEDs needed in the attempt to control the epilepsy [12]. In addition to this, the risk of adverse events and rebound phenomena can be different in relation to administered AEDs [6], but we still lack an easily quantifiable seizure prediction indicator to control the occurrence of spontaneous recurrent seizures (SRSs) during the presurgical evaluation of patients with refractory epilepsy.

Among other AEDs, (*S*)-α-ethyl-2-oxopyrrolidine acetamide (levetiracetam, LEV) [13] is commonly used in clinical practice. Moreover, LEV has already been tested in different animal models of seizures and epilepsy, in order to evaluate its anticonvulsant effects and tolerability [14,15,16]. Indeed, it has been demonstrated that LEV significantly reduces the seizure frequency during 2 weeks of treatment in pilocarpine-treated rats. However, the individual response of animals to LEV varies from complete seizure control to no effect, even if plasma drug levels are well within the therapeutic range [14].

LEV has the advantage of being water-soluble and thus can be administered without using any solvent potentially able to affect the central nervous system function. To this regard, LEV appears as an ideal tool to test the rebound effect in a post-*status epilepticus* model of chronic epilepsy [17]. Thus, we decided to use LEV and designed an experiment to fully characterize its therapeutic properties and the consequences of LEV rapid withdrawal. To this aim, kainic acid (KA)-treated rats were first monitored by video-electrocorticographic (v-ECoG) recordings coupled with power band spectrum analysis before, during LEV administration via osmotic minipumps for a week, and subsequently in the weeks following LEV withdrawal. In view of our previous finding indicating a selective reduction of allopregnanolone levels in the hippocampus of KA-treated rats, in which this neurosteroid was related to the seizure occurrence observed in epileptic animals [18], we determined whether allopregnanolone and other neurosteroids could be altered after LEV treatment, using liquid chromatography-electrospray-tandem mass spectrometry (LC-ESI-MS/MS). Indeed, neurosteroids have been implicated in a phenomenon similar to the rebound effect in epileptic female rats treated with progesterone for a week, in which a 2-fold increase in the seizure frequency occurred in 57% of animals in the week following progesterone withdrawal. Notably, this effect was independent of progesterone receptor activation and probably was due to the conversion of progesterone to other neurosteroids [19].

## 2. Results

### 2.1. Different Responses to Treatment

During LEV treatment, seven out of 15 rats (47%) presented a complete response consisting of the suppression of SRSs during the overall period of observation. The remaining rats (53%, eight out of 15 rats) experienced an incomplete response to LEV. By comparing the occurrence of SRSs under LEV administration to that observed in the pre-treatment week of the same animals (during which all rats displayed at least one SRS), we observed a significant difference (*p* = 0.006, Fisher’s exact test) suggesting that LEV efficaciously suppressed ictogenesis. In the same time interval, 93% of epileptic animals (14 out of 15 rats) receiving saline displayed SRSs (*p* = 0.035 vs. LEV).

### 2.2. Characterization of the Duration of SRSs

**Figure 1:**
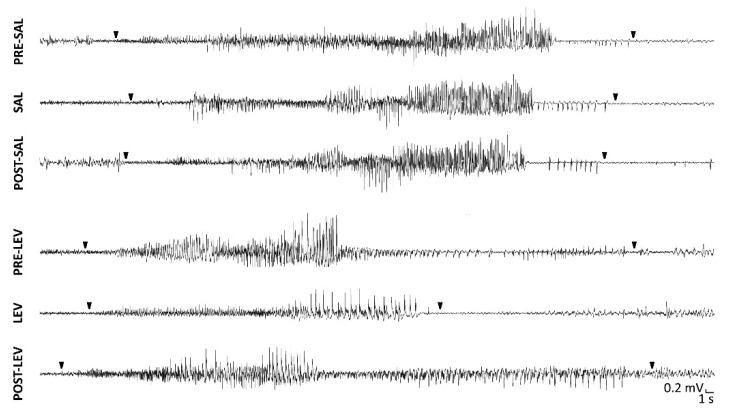
Spontaneous recurrent seizures (SRSs) after induction of *status epilepticus* in epileptic rats treated with levetiracetam (LEV) or saline. SRSs were recorded in epileptic rats during the treatment with LEV or saline, but also 1 week before and 1 week after LEV or saline administration by subcutaneous osmotic minipumps. Note that SRSs appeared in incomplete responders to LEV. Arrowheads point to onset and termination of SRSs. PRE-, before osmotic minipump implantation; POST-, after minipump implantation; SAL, saline.

By visual inspection, SRSs recorded in LEV-treated rats which did not maintain an adequate drug response appeared to differ (i.e, duration and postictal ECoG) from those observed during the week preceding the implantation of minipumps (PRE-LEV and PRE-SALINE), and from those characterized in epileptic rats treated with saline in the same period, as well as in the following week (POST-SALINE). Moreover, SRSs recorded during the treatment with LEV also seemed to have different features, in terms of duration and postictal ECoG, in comparison to those that developed after the treatment (POST-LEV) (Figure 1). This is in agreement with the literature suggesting that relapsing seizures are related to specific ictal and interictal activity [20].

**Figure 2:**
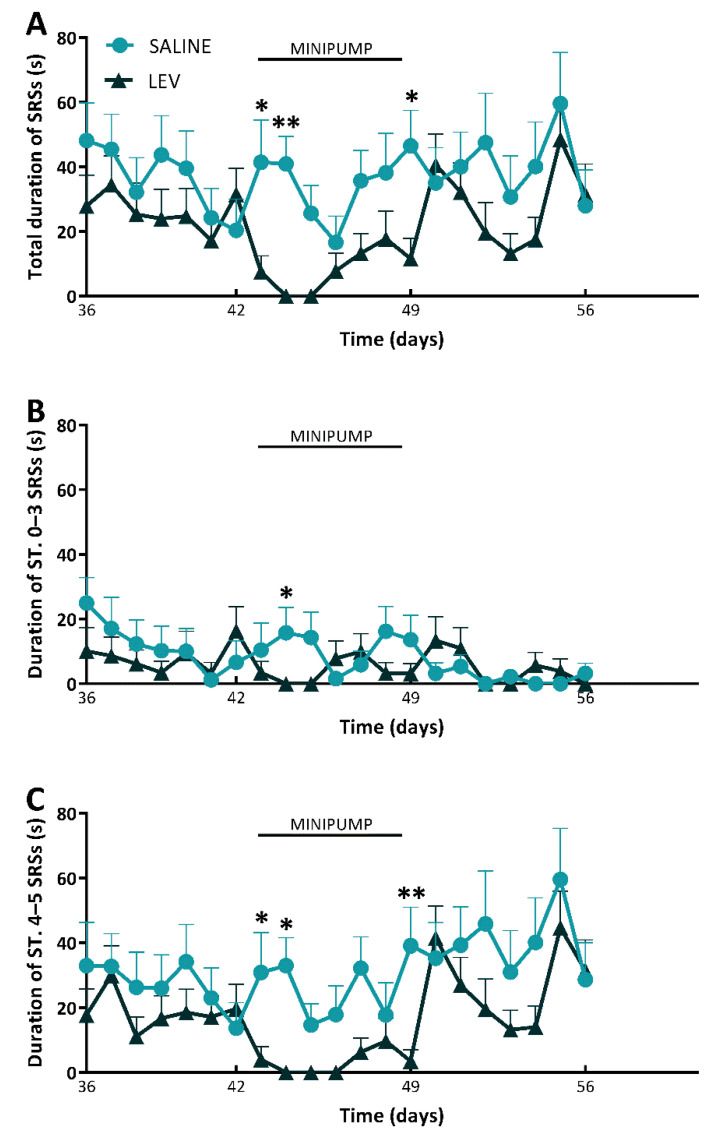
Duration of spontaneous recurrent seizures (SRSs) in epileptic rats treated with levetiracetam (LEV) or saline. All epileptic rats (*n* = 30) were treated with LEV (*n* = 15) or saline (*n* = 15). In (**A**), the treatment with LEV had beneficial effects on total duration of SRSs 1, 2 and 7 days after the implantation of osmotic minipump. Particularly, LEV displayed significant effects on stage 0–3 SRSs (**B**), and generalized stage 4–5 SRSs (**C**). * *p* < 0.05, ** *p* < 0.01, Holm-Šidák test; SALINE vs. LEV. ST., stage.

After the Shapiro-Wilk test (*p* = 0.254), data were analyzed by 2-way (treatment x time) repeated measures analysis of variance (ANOVA). This analysis showed a statistically significant effect of treatment for daily duration of all SRSs (F(1279) = 14.10, *p* = 0.002), paralleled by a main effect of time for daily duration of all SRSs (F(20,279) = 2.13, *p* = 0.004). However, there was no statistically significant interaction between treatment and time for daily duration of all the different types of SRSs (nonconvulsive and convulsive) in rats (F(20,279) = 0.89, *p* = 0.595), indicating that LEV effects were not dependent on changes in this seizures’ feature by time. Precisely, the total duration of SRSs was reduced by LEV in comparison to saline during the first two days of treatment (*p* = 0.018 for the 1st day, *p* = 0.004 for the 2nd day; Holm–Šidák test). LEV also decreased the total duration of SRSs at approximately 30% of control levels in the last day of treatment (SALINE vs LEV, *p* = 0.028) (Figure 2A). Interestingly, these differences were found on the 2nd day of treatment for nonconvulsive, stage 0–3 SRSs (*p* = 0.04, SALINE vs. LEV) (Figure 2B), and on the 1st (*p* = 0.048), 2nd (*p* = 0.016) and 7th day (*p* = 0.005) for convulsive, generalized tonic-clonic SRSs (Figure 2C).

### 2.3. Characterization of SRSs Occurrence

**Figure 3:**
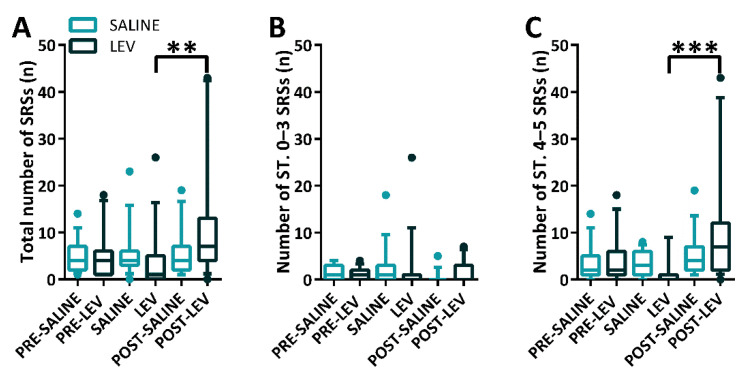
Weekly occurrence of spontaneous recurrent seizures (SRSs) in epileptic rats treated with levetiracetam (LEV) or saline. All rats (*n* = 30) were treated with LEV (*n* = 15) or saline (*n* = 15). In (**A**), the discontinuation of LEV significantly induced the rebound effect. In (**B**), LEV displayed no significant effects on stage 0–3 SRSs. In (**C**), LEV significantly induced rebound phenomena of stage 4–5 SRSs after its discontinuation. The whiskers of the boxes represent the 10–90th percentile. ** *p* < 0.01, *** *p* < 0.001, Tukey’s test, LEV vs. POST-LEV. ST., stage.

After the significant result (*p* < 0.05) of the Shapiro-Wilk test, data were analyzed by Friedman repeated measures ANOVA on ranks. To this aim, we considered all seizures occurring in the different treatment conditions (i.e., preceding the minipump implantation, during saline or LEV delivery, and after removal of minipumps), as illustrated in Figure 3. This analysis revealed that differences in the median values among the treatment groups were statistically different (*p* = 0.01), when considering the weekly occurrence of all SRSs types. Then, the multiple comparison procedure (Tukey’s test) showed that the rapid LEV discontinuation induced an abrupt increase in the weekly occurrence of all generalized SRSs, up to 700% of previous median levels in the LEV-treated group (LEV vs POST-LEV, *p* = 0.002, Figure 3A). Interestingly, these changes did not occur for stage 0–3 SRSs (Figure 3B) but involved only tonic-clonic SRSs (LEV vs POST-LEV, *p* < 0.001, Figure 3C).

### 2.4. Relationship between Frontal Power Band Spectrum and SRSs in the Week Following the Treatment

A total of 26 out of 30 epileptic animals (SALINE = 13, LEV = 13) were analyzed to determine a linear relationship between the frontal power band spectrum of postictal ECoG before and after the treatment (the independent variable), and the total number of SRSs after the treatment (the dependent variable). Two animals (SALINE = 1, LEV = 1) were excluded because their frontal electrodes were damaged (only occipital electrodes were useful to detect v-ECoG signals), whereas two other animals (SALINE = 1, LEV = 1) were excluded because they did not develop SRSs in the week following the treatment.

**Figure 4:**
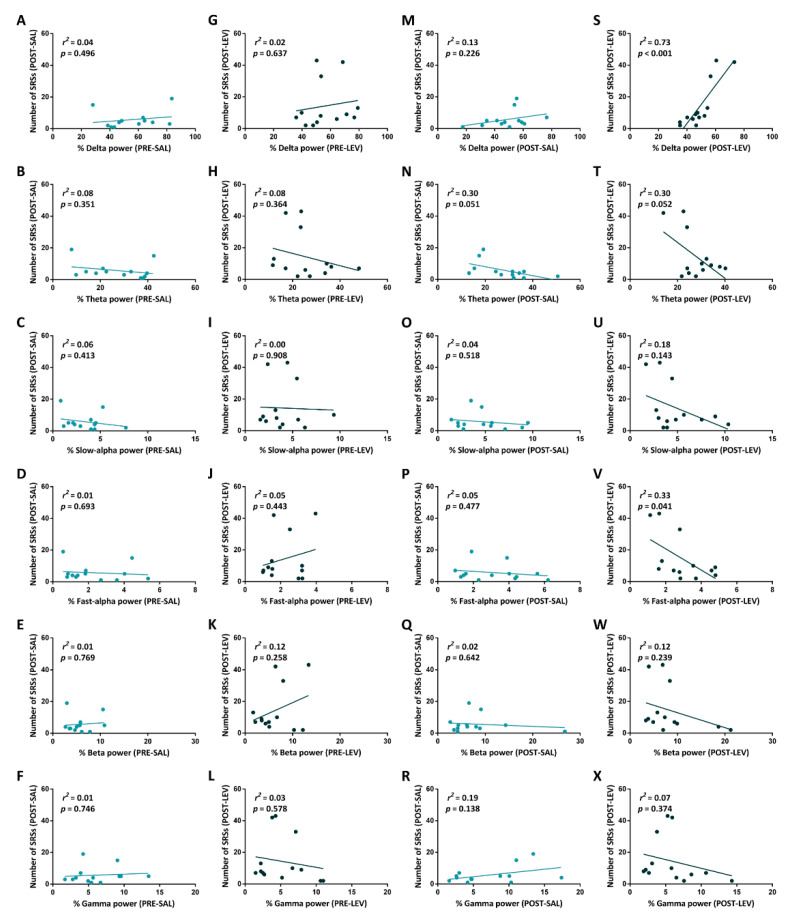
Relationship between power band spectrum and spontaneous recurrent seizures (SRSs) following treatment with levetiracetam (LEV) or saline. Epileptic animals treated with LEV (*n* = 13) and epileptic rats treated with saline (*n* = 13) were used to evaluate whether a relationship existed between changes in the power band spectrum during the postictal events, and the number of SRSs in the week following the treatment. The relationship (*r*^2^) between the frontal power band spectrum in the pre-treatment interval and the number of SRSs in the week following saline administration were determined as in (**A**–**F**). Relationship between the frontal power band spectrum in the pre-treatment interval and the number of SRSs in the week following LEV treatment were shown in (**G**–**L**). Similarly, relationship between the frontal power band spectrum in the post-treatment interval and the number of SRSs in the week following saline administration were illustrated in (**M**–**R**). Finally, relationship between the frontal power band spectrum in the post-treatment interval and the number of SRSs in the week following LEV administration were illustrated in (**S**–**X**). Statistical analysis was performed using a linear regression analysis with number of seizures being the dependent and the frontal power band spectrum as the independent variable. PRE-, before osmotic minipump implantation; POST-, after minipump implantation; SAL, saline.

We did not find any linear relationship between: (i) the frontal power band spectrum in the pre-treatment interval, and the number of SRSs in the week following saline administration (Figure 4A–F); (ii) the frontal power band spectrum in the pre-treatment interval, and the number of SRSs in the week following LEV administration (Figure 4G–L); and (iii) the frontal power band spectrum in the post-treatment interval, and the number of SRSs in the week following saline administration (Figure 4M–R). Interestingly, the power of delta was linearly related to the number of SRSs after LEV administration (*r*^2^ = 0.73, *p* < 0.001) (Figure 4S). At variance, a weaker, inverse relationship was found between the power of fast-alpha, and the number of SRSs after LEV administration (*r*^2^ = 0.33, *p* = 0.041) (Figure 4V). Finally, no relationship was found between the frontal power of theta, slow-alpha, beta, and gamma in the post-treatment interval, and the number of SRSs in the week following LEV administration (Figure 4T,U,W,X).

### 2.5. Levels of Neurosteroids in the Hippocampus and Neocortex of Epileptic Rats, Measured 2 Weeks Following the Treatment

The levels of pregnenolone sulfate, pregnenolone, progesterone, 5α-dihydroprogesterone, allopregnanolone, and pregnanolone were determined in both the hippocampus (SALINE = 8, LEV = 11) and neocortex (SALINE = 8, LEV = 13) of 21 epileptic rats, 2 weeks after treatment (64 days after KA-induced *status epilepticus*). Particularly, two hippocampi were excluded from the analysis, because of technical issues with the sample storage. Moreover, values under the limit of quantification (LOQ) and outliers, identified by Grubbs’ test, were excluded from the statistical analysis. Additionally, the daily frequency of seizures in these animals in the week preceding the killing was significantly higher in the LEV group (POST-SALINE: 0.50, 0.50–0.67, median value and interquartile range; vs. POST-LEV: 3.79, 0.59–8.375; *p* < 0.040, Mann-Whitney test).

Briefly, pregnenolone sulfate was significantly reduced in the hippocampus (*p* = 0.001) and neocortex (*p* = 0.009) of epileptic rats treated with LEV, in comparison to the epileptic animals treated with saline. The levels of pregnenolone and progesterone did not differ significantly in both hippocampus and neocortex. Furthermore, 5α-dihydroprogesterone was significantly reduced in the hippocampus (*p* = 0.018), but not in the neocortex of LEV-treated rats, compared to saline-treated rats. At variance, allopregnanolone was significantly increased in the neocortex (*p* = 0.034), but not in the hippocampus of LEV-treated rats. Additionally, pregnanolone was significantly reduced in the neocortex (*p* ≤ 0.001), but not in the hippocampus, of LEV-treated rats (Table 1).

**Figure 5:**
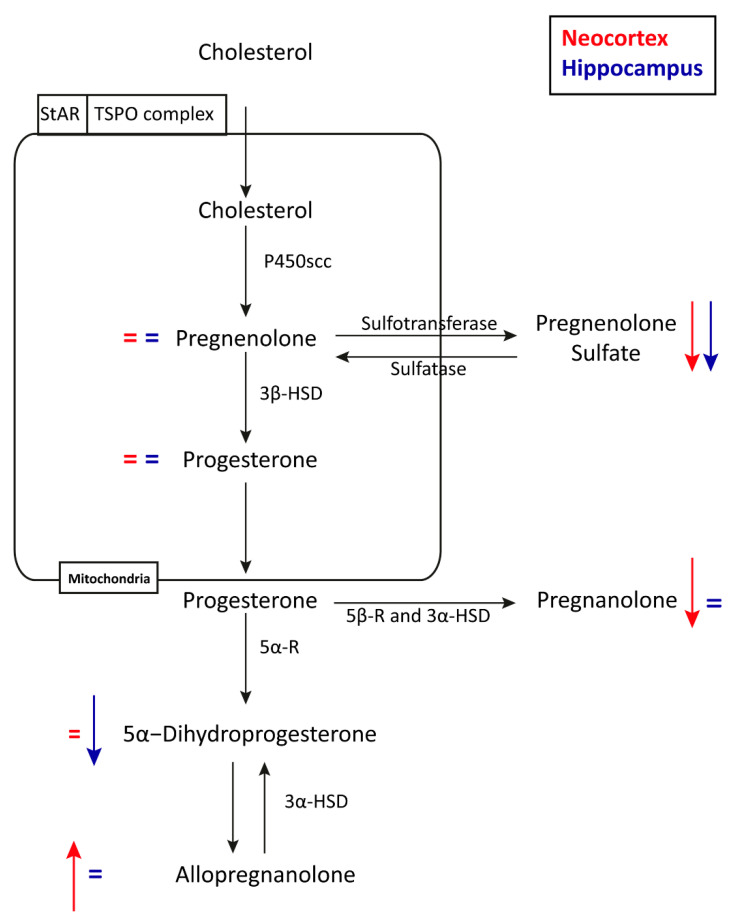
Diagram of neurosteroid synthesis and levels of neurosteroids in epileptic rats treated with levetiracetam (LEV) or saline. Changes in neurosteroids’ neocortical levels of LEV-treated rats are illustrated by red arrows, whereas changes in neurosteroids’ hippocampal levels of LEV-treated rats are illustrated by blue arrows. StAR, steroidogenic acute regulatory protein; TSPO, translocator protein; P450scc, cytochrome P450-associated cholesterol side chain cleavage enzyme; 3α-HSD, 3α-hydroxysteroid dehydrogenase; 3β-HSD, 3β-hydroxysteroid dehydrogenase/Δ^5-4^ isomerase; 5α-R, 5α-reductase; 5β-R, 5β-reductase.

In summary, in comparison to epileptic animals treated with saline, pregnenolone sulfate was the only analyte reduced both in the hippocampus and neocortex of epileptic animals treated with LEV. At variance, levels of pregnanolone and allopregnanolone were significantly changed only in the neocortex, whereas those of 5α-dihydroprogesterone were significantly reduced only in the hippocampus of LEV-treated rats (Figure 5).

### 2.6. Relationship between the Number of SRSs after Treatment and Levels of Hippocampal Neurosteroids

In view of our previous findings on the relationship between allopregnanolone hippocampal levels and seizure occurrence in epileptic rats [18], we also analyzed the linear relationship between the number of SRSs after treatment with LEV (the independent variable) and hippocampal levels of allopregnanolone in LEV-treated rats (the dependent variable). Particularly, we evaluated the possible relationship between the number of SRSs after treatment with LEV (8 weeks after treatment with KA) and levels of various neurosteroids in the hippocampus.

**Figure 6:**
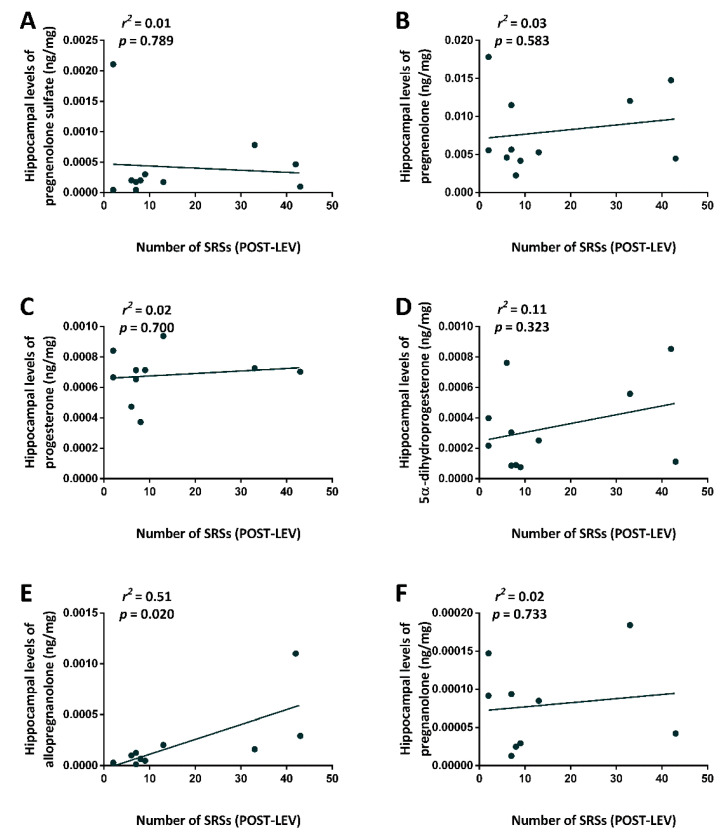
Relationship (*r*^2^) between the number of spontaneous recurrent seizures (SRSs) after treatment with levetiracetam (LEV, 8 weeks after treatment with KA) and the hippocampal neurosteroid levels measured in LEV-treated rats, 2 weeks after treatment. Statistical analysis was performed using a linear regression analysis, with neurosteroid levels as dependent variable and the number of SRSs as independent variable (**A**–**F**). POST-, after minipump implantation.

No linear relationship was found between the number of SRSs at 8 weeks after treatment with KA and the hippocampal levels of pregnenolone sulfate (*r*^2^ = 0.01, *p* = 0.789) (Figure 6A), pregnenolone (*r*^2^ = 0.03, *p* = 0.583) (Figure 6B), progesterone (*r*^2^ = 0.02, *p* = 0.7) (Figure 6C), 5α-dihydroprogesterone (*r*^2^ = 0.11, *p* = 0.323) (Figure 6D), or pregnanolone (*r*^2^ = 0.02, *p* = 0.733) (Figure 6F). At variance, the number of SRSs was positively related to hippocampal levels of allopregnanolone after treatment with LEV (*r*^2^ = 0.51, *p* = 0.02) (Figure 6E).

### 2.7. Relationship between the Power of Delta Band in the Postictal Component of Seizures and Levels of Hippocampal Neurosteroids

**Figure 7:**
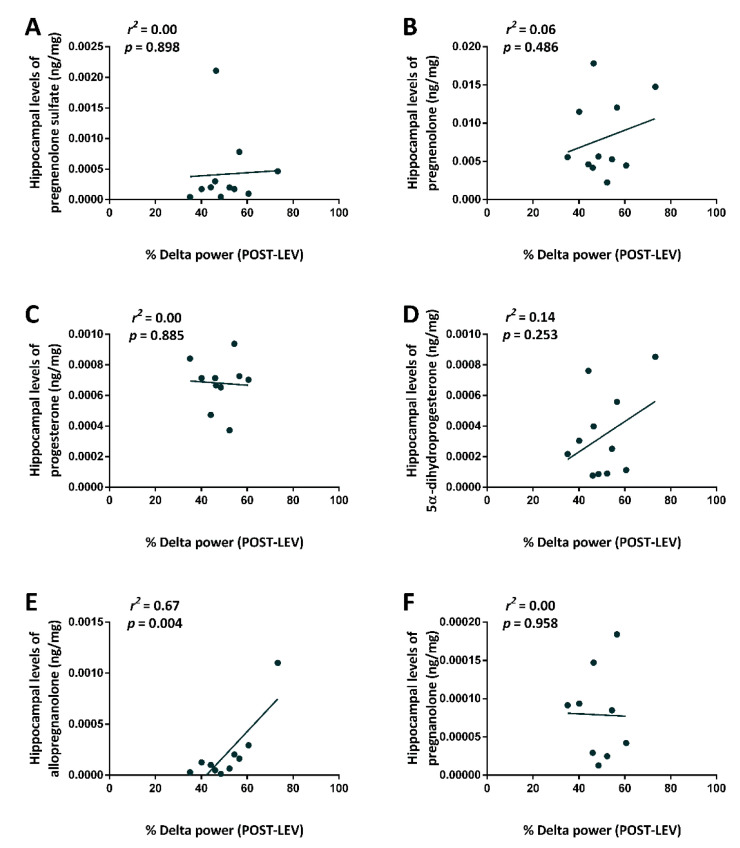
Relationship (*r*^2^) between the frontal power band spectrum (% Delta power) in the post-treatment interval and the hippocampal neurosteroid levels measured after treatment with levetiracetam (LEV), 2 weeks after treatment. Statistical analysis was performed using a linear regression analysis, with neurosteroid levels as dependent variable and the frontal power band spectrum as independent variable (**A**–**F**). POST-, after minipump implantation.

As we found a relationship between hippocampal allopregnanolone levels and SRSs, we also evaluated the possible linear relationship between the percentage of delta power of postictal ECoG after treatment with LEV (the independent variable) and levels of various neurosteroids in the hippocampus (the dependent variable). No linear relationship was found between the power of delta in postictal traces and hippocampal levels of pregnenolone sulfate (*r*^2^ = 0.0, *p* = 0.898) (Figure 7A), pregnenolone (*r*^2^ = 0.06, *p* = 0.486) (Figure 7B), progesterone (*r*^2^ = 0.0, *p* = 0.885) (Figure 7C), 5α-dihydroprogesterone (*r*^2^ = 0.14, *p* = 0.253) (Figure 7D), or pregnanolone (*r*^2^ = 0.0, *p* = 0.958) (Figure 7F). At variance, the power of delta was related to hippocampal levels of allopregnanolone (*r*^2^ = 0.67, *p* = 0.004) (Figure 7E).

## 3. Discussion

In the present study, LEV administration resulted in the following major effects: (i) SRSs were efficaciously suppressed in the majority of rats during the week of drug treatment, and the duration of SRSs was reduced in rats partially responding to LEV, being this result significant in both nonconvulsive and convulsive SRSs; (ii) we observed a remarkable rebound effect after abrupt LEV withdrawal; (iii) the delta power in postictal recordings was linearly related to the seizure occurrence in the previously LEV-treated rats, thus suggesting the involvement of this electrographic component in the rebound effect. Additionally, we found that hippocampal levels of allopregnanolone were related to delta power in postictal recordings of epileptic rats.

A required premise for our experiment was the confirmation of the anticonvulsant effects of LEV. To this aim, we adopted the systemic KA model of chronic epilepsy [17], by introducing substantial differences with respect to most of the published research on testing AEDs in the same animal model. With few exceptions [21], the previous experiments did not address the frequency of nonconvulsive SRSs or the overall seizure duration, but only the frequency of convulsive SRSs was monitored by behavioral analysis [22]. Moreover, topiramate, carbamazepine, and carisbamate, but not LEV were the examined AEDs after the systemic injection of KA [23,24,25]. Particularly, repeated intraperitoneal injections of topiramate (0.3–100 mg/kg) or carisbamate (0.3–30 mg) resulted in a dose-dependent effect on convulsive SRSs, though carisbamate appeared more efficacious than topiramate in suppressing convulsive SRSs [23,25]. Also intraperitoneal and oral administration of carbamazepine (respectively, 30 and 100 mg/kg) effectively reduced the frequency of motor SRSs [24]. In additional experiments, carbamazepine (30 mg/kg) significantly reduced the frequency of convulsive SRSs without affecting nonconvulsive SRSs recorded in the dentate gyrus. Carbamazepine adequately suppressed the frequency of all SRSs only at 100 mg/kg, while duration remained unchanged also at this dose [21]. Thus, LEV in our animals produced effects comparable to those of the other AEDs.

Similarly, an important achievement of our study was the possibility to reproduce the rebound effect due to rapid AED withdrawal, in order to provide a ground on which to characterize the frequently observed consequences of presurgical evaluation of patients screened for surgical resection of epileptic foci [5]. In clinical practice, AED discontinuation is required during a long EEG monitoring period to enhance the rate of successful diagnostics [7] and to precisely defining the epileptogenic zone [26,27]. However, an important concern is that few guidelines on AED withdrawal during a lengthy stay in EEG monitoring have been published [8], so the outcome might be doubly uncertain. Indeed, this procedure may trigger secondarily generalized tonic-clonic SRSs, SRS clustering, and *status epilepticus* in patients [9,28]. Different dynamics could be responsible for these consequences. For instance, a previous poor response to drug treatment seemed to induce severe SRSs in patients, after carbamazepine withdrawal [5,29]. In monotherapy, one explanation for the increased seizure frequency above pre-AED values upon discontinuation of treatment could be the development of tolerance to the antiseizure effect of LEV, which was a mechanism described by several groups [15,30,31]. Particularly, tolerance to LEV could be a result of functional adaptation of LEV’s target(s) to the presence of the AED, which might result in increased seizure frequency after AED discontinuation. However, in the attempt to define a risk index for the rebound effect after rapid AED withdrawal, our animal model could be useful [17,18,32], since our animals developed an abrupt increase of generalized tonic-clonic SRSs, after LEV discontinuation. Alternatively, other mechanisms could contribute to the rebound effect, in particular the time window in which specific brain areas might be interested by neurogenesis [33]. In this regard, it was demonstrated that kindling with pentylenetetrazole administration for approximately 25 days led to a remarkable increase in seizure susceptibility which was coincident with the timing required for newborn neurons to differentiate and integrate into the hippocampal network [34], a phenomenon which could contribute to the time-dependent increase in SRSs of KA-treated rats in a manner independent of AED administration.

In our study, we found the delta power band spectrum of the postictal ECoG increased in presence of a higher occurrence of generalized SRSs, supporting the view that delta oscillations are important for ictogenesis. In clinical studies, power changes in specific frequency bands demonstrated their potentiality as seizure prediction indicators [35]. LEV, in turn, appeared to be effective in decreasing epileptiform EEG abnormalities. In drug-naïve epilepsy patients, LEV monotherapy was reported to have a region-specific spectral effect on the background EEG activity by decreasing slow frequency power and increasing fast frequency power [36]. In addition to this, the relevance of the immediate postictal EEG was long-debated and became of interest when it was found to be useful to localize the epileptic zone, at least in some type of epilepsies [37,38]. In comparison with the ictal EEG, the postictal EEG is less modified by muscle and movement artifacts, and allows the analysis of a longer period of EEG recording [37]. Interestingly, SRSs were followed by postictal EEG changes in more than 70% of patients affected by temporal lobe epilepsy, and regionally accentuated delta slowing was the most frequent postictal change reported after SRSs [39]. Furthermore, in a cohort study of patients suffering from temporal lobe epilepsy, it was demonstrated that the relative spectral delta power during postictal EEG increased more in secondary generalized seizures than in simple partial seizures, so to appear strictly related to the increased severity of seizures [40]. In patients with a history of uncontrollable generalized tonic-clonic SRSs, and mainly those at greatest risk for sudden unexpected death in epilepsy, it was observed that low delta (0.5–1.5 Hz) and gamma (30–50 Hz) coupled signals were remarkably increased in the postictal generalized EEG suppression state, as compared to baseline. In general, these changes in neuronal oscillations were consistent in the postictal suppression state, regardless of whether the suppression was generalized or not across channels. Then, it was also suggested that very high frequency oscillations (600–2000 Hz) could be combined with low delta as an additional biomarker for postictal states [41].

We explored neurosteroid levels in the brain as a possible mechanism involved in the rebound effect, since it has been reported that administration of progesterone is followed by a rebound effect with the 2-fold increase in seizure frequency after abrupt interruption of treatment [19]. This suggested that changes in brain neurosteroid levels could be accompanied by corresponding alterations in the seizure occurrence. Indeed, in a previous study [18] we found a significant relationship between the hippocampal levels of allopregnanolone and seizures occurring in chronic epileptic rats, a phenomenon that we interpreted as the activation of an anticonvulsant compensatory mechanism [18]. Here, we confirmed the significant relationship between allopregnanolone levels and seizure occurrence in the hippocampus of LEV-treated rats, but we found an even more remarkable relationship between the power of delta oscillations in postictal traces and hippocampal levels of allopregnanolone. Since delta activity was also related to the seizure frequency in LEV-treated rats, allopregnanolone could have played a role in the rebound effect, which should be further investigated with specific pharmacological approaches aimed at inhibiting allopregnanolone production (i.e., finasteride) [42].

Additionally, we found various changes in neurosteroid levels in both neocortex and hippocampus by comparing saline and LEV-treated rats. Especially, the most consistent alteration was the remarkable reduction in pregnenolone sulfate levels found in both brain regions of LEV-treated rats. However, pregnenolone sulfate is a proconvulsant [42] and its reduction could be hardly related to the observed rebound effect. The anticonvulsant neurosteroid pregnanolone also was dramatically reduced in the neocortex of LEV-treated rats but not in the hippocampus. Moreover, allopregnanolone, which acts similarly to pregnanolone as a positive modulator of inhibitory currents [43], was moderately but significantly increased in the neocortex of LEV-treated rats, maybe playing a compensatory role for the reduction in pregnanolone levels. Overall, these changes in neocortical neurosteroid levels appear to be less relevant to understand the complex dynamics of the rebound effect, for which the hippocampal neurosteroid levels were apparently more critical.

## 4. Materials and Methods

### 4.1. Animals

The study protocol was authorized by the Italian Ministry of Health (323/2015-PR), after approval by the university Animal Welfare Body. All experiments were performed according to the European Directive 2010/63/EU and the consequent Italian act (DM 26/2014). Adult male Sprague-Dawley rats (Charles River, Calco, Italy) were housed in a specific pathogen-free facility with a controlled environment and ad libitum access to water and food. A total of 30 rats, with initial weights of 175–200 g, were used in this study. All efforts were done to refine procedures and protect the animals’ welfare.

### 4.2. Experimental Design

Rats were implanted with electrodes, treated with an intraperitoneal injection of KA (15 mg/kg; Sigma-Aldrich, Milan, Italy) to induce a *status epilepticus* one week after surgery, and given a subcutaneous injection of Ringer’s lactate solution (3–5 mL) along with softened rat chow at the end of *status epilepticus* to minimize discomfort.

After 6 weeks from *status epilepticus* induction, rats were anesthetized with volatile isoflurane in order to implant subcutaneously a minipump delivering a continuous dosing over one week (2ML1 ALZET, flow rate: 10 µL/h, DURECT Corporation, Cupertino, CA, USA). Rats were randomly divided into two groups: the first group consisted of 15 epileptic rats that had a minipump able to deliver LEV (generously provided by UCB Pharma, Brussels, Belgium) dissolved in saline at 300 mg/kg/day [44]; the second group, control group, consisted of 15 epileptic rats with a minipump delivering saline. The duration of the *status epilepticus* was considered in the randomization approach to guarantee that important baseline levels did not differ between the LEV group and saline control group. Particularly, *status epilepticus* lasted for 9.51 ± 29.12 h in the saline-treated group and 10.31 ± 57.56 h in the LEV-treated group (*p* < 0.602; SALINE vs. LEV), respectively. In all animals, SRSs were continuously monitored. Specifically, a blind analysis of all video-ECoG recordings belonging to saline- and LEV-treated groups were analyzed by three different expert raters. The postictal events were then collected and evaluated by a blind power spectrum analysis. The levetiracetam withdrawal was defined as “rapid” because we completely discontinued the levetiracetam without a gradual reduction in dosage.

### 4.3. v-ECoG Recordings

As described previously [45], rats were implanted with epidural electrodes in order to continuously record ECoG data from frontal and occipital cortices. More precisely, guiding holes were drilled and epidural electrodes (stainless steel Ø = 1 mm; PlasticsOne, Roanoke, VA, USA) were implanted in frontal (bregma 0 mm, 3.5 mm lateral from midline) and occipital cortices (bregma −6.5 mm, 3.5 mm lateral from midline) of both hemispheres. One electrode was implanted below the lambda on the midline in all rats and used as a reference. Electrodes were connected through steel wire to terminal gold pins (Bilaney Consultant GmbH, Düsseldorf, Germany) inserted in a plastic pedestal (PlasticsOne) cemented on heads. The four recording electrodes were used for video-ECoG monitoring, whereas only frontal electrodes were used for power band analysis. Volatile isoflurane was used during electrode implantation to induce a deep anesthesia, assessed by deep breath, loss of tail and eye reflexes. Gel containing 2.5 g lidocaine chloride, 0.5 g neomycin sulfate and 0.025 g fluocinolone acetonide (Neuflan^®^ gel; Molteni Farmaceutici, Scandicci, Italy) was applied at the end of the surgery to reduce acute pain and risk of infection. All rats were monitored until complete recovery from anesthesia and housed in single cages with no grids or environmental enrichments to avoid risk of headset loss. The EcoG was recorded via cable connection between headset and preamplifiers. Electrical activity was digitally filtered (0.3 Hz high-pass, 500 Hz low-pass), acquired at 1 kHz per channel, and stored on a personal computer after the mathematical subtraction of traces of recording electrodes from trace of reference electrode, using a PowerLab8/30 amplifier connected to 4 BioAmp preamplifiers (AD Instruments; Dunedin, Otago, New Zealand). Videos were digitally captured through a camera connected to the computer and synchronized to the ECoG traces through LabChart 8 PRO internal trigger.

### 4.4. Behavioral and ECoG Analysis

ECoG traces were digitally filtered offline (band-pass: high 50 Hz, low 1 Hz) and manually analyzed using LabChart 8 PRO software (AD Instruments) by expert raters. *Status epilepticus* was defined as the period of time in which rats either did not recover normal behavior between a seizure and the other, or in which they displayed continuous shaking for more than 5 min. The end of *status epilepticus* was characterized by a progressive reduction in frequency of the continuous electrographic spikes, preceding a silent period. Moreover, the termination of *status epilepticus* was accompanied by recovery of normal behavior. In our animal model, *status epilepticus* was allowed to self-terminate [17].

Seizures were defined as ECoG segments with a minimum duration of 10 s, continuous synchronous high-frequency activity, and an amplitude of at least twice the previous baseline [17]. They were also screened for the presence of a postictal depression, that was the period of time in which the brain recovered from the seizure before regaining normal function. Seizures and their durations were determined in the ECoG traces, and then investigated for related behavior [46] by using the synchronized video recordings. In particular, all seizures were scored as stage 0 (or subclinical) if a clear epileptiform ECoG signal was observed without corresponding evident behavior in the video; stage 1–2 in the presence of absence-like immobility, “wet-dog shakes”, facial automatisms, and head nodding; stage 3, when presenting with forelimb clonus and lordosis posture; stage 4, corresponding to generalized seizures and rearing; and stage 5, when seizures consisted of rearing with loss of posture and/or wild running, followed by generalized convulsions. The initiation of the first SRS was defined by a single, large ECoG spike, followed by large high-frequency activity. The progression was characterized by individual spike-like events followed by regular, large, high-frequency events. At the end of this activity, large-amplitude waves with multiple superimposed spike-like events were followed by a silent period [47]. Artifacts were carefully removed from the v-ECoG analysis of SRSs. They were identified as (i) high frequency signals associated with masticatory movements in the video recording, (ii) interference appearing as a thickening caused by superposition of 50 Hz mains in the ECoG, and (iii) electrode or cable-related technical artifacts.

Changes in the brain oscillations during 10 s of postictal ECoG were analyzed in the frontal cortex. Indeed, given the various alterations that could lead to SRSs, icto- and epileptogenic mechanisms ultimately appeared to converge on a set of large-scale neural networks, in which thalamus and midline frontal and parietal cortices could play an important role, even if they did not contain the seizure focus [48]. In particular, EDFbrowser (1st order butterworth high-pass filter: 1 Hz; powerline interference removal: 50 Hz) [49] was used to determine a relative indication of the distribution of power over the frequency regions ranging from 1 to 100 Hz, expressed in percentage (%) and recorded from the frontal electrodes. Thus, the power band spectrum analysis included delta (δ, 0–4 Hz), theta (θ, 4–8 Hz), slow-alpha (α, 8–10 Hz), fast-alpha (α, 10–12 Hz), beta (β, 12–24 Hz), and gamma (γ, 24–100 Hz) frequencies in 10-s epochs on a continuous ECoG.

### 4.5. Quantitative Analysis of Neurosteroids by LC-ESI-MS/MS

Rats were euthanized 64 days after the treatment protocol. Up to 21 rats treated with LEV (*n* = 13) or saline (*n* = 8) were used. Brains were carefully removed after euthanasia (isoflurane) and chilled on ice to dissect both hippocampi and neocortices for LC-ESI-MS/MS analysis. Details about chemicals, reagents and standard solutions were published previously [18,50].

All samples were spiked with an internal standard solution, vortexed and added with acetonitrile/methanol (70/30; +1.0% formic acid). The samples were sonicated, centrifuged, and the obtained supernatants were purified on Phree–SPE cartridges to remove endogenous phospholipids. Eluates were concentrated, derivatized with Amplifex Keto Reagent and transferred to autosampler vials for LC-ESI-MS/MS analysis, which was performed on a Kinetex XB–C18 column (100 × 2.1 mm; 2.6 µm particle size) preceded by a C18 Security Guard cartridge (2.1 mm) (Phenomenex; Torrance, CA, USA). Mass spectrometric detection was performed using a QQQ–MS/MS (6410B) triple quadrupole system (Agilent; Waldbronn, Germany) operating in electrospray positive ionization mode. The values for the limit of quantification (LOQ) for each analyte were calculated from the calibration plot as 10σ/S, where σ and S are the standard deviation and the slope of the regression line. Particularly, the values for the LOQ were 0.0000058 ng/mg for pregnenolone sulfate, 0.0000011 ng/mg for pregnenolone, 0.0000065 ng/mg for progesterone, 0.0000098 ng/mg for 5α-dihydroprogesterone, and 0.0000029 ng/mg for allopregnanolone and pregnanolone.

### 4.6. Statistics

The sample size of 30 animals was calculated by performing a power analysis (G*power 3.1.9.2), which considered an effect size of 0.183, alpha-error of 0.002, a power (1-ß) of 0.80, and some dropouts from the study. The number of animals was supported by our published data [17]. In the LEV-treated group, we compared the percentage of seizure occurrence at 13 (PRE-minipump) and at 14 (minipump) weeks of age by using the Fisher’s exact test, which is appropriate for small samples. Using the same test, we also compared the percentage of animals with seizures during the treatment period in LEV- and saline-treated groups. After having tested for normality of distribution (Shapiro-Wilk test), data on seizure duration were analyzed by a 2-way (Treatments: LEV, SALINE) × 21 (Time Intervals expressed in days: 36, 37, 38, 39, 40, 41, 42, 43, 44, 45, 46, 47, 48, 49, 50, 51, 52, 53, 54, 55, 56) repeated measures ANOVA. Post hoc comparisons, when appropriated, were done using a multiple comparison test (Holm-Šidák). Data on seizure occurrence were analyzed by Friedman repeated measures ANOVA on Ranks, followed by the Tukey test. Data on weekly frequency (16 weeks of age) were analyzed by Mann-Whitney test. For analysis of the neurosteroid levels, treatment groups (saline and LEV) were compared by the Student’s t-test. Values under LOQ and outliers, identified by Grubbs’ test, were excluded. Linear regression (expressed with r-square and *p*-value) was used to model the relationship between the number of SRSs (dependent variable) and the power band spectrum variables expressed in percentage (independent variables). Similarly, linear regression was adopted to define the relationship between the hippocampal levels of neurosteroids and the number of SRSs 8 weeks after KA-induced SE in LEV-treated rats. Moreover, linear regression was also used to model the relationship between the hippocampal levels of neurosteroids and the power band spectrum variables expressed in percentage. All values are presented as medians and interquartile ranges or mean ± standard error of the mean (SEM), with *p* < 0.05 having been chosen as the significant difference level. Statistical analyses were performed with SigmaPlot 13 (Systat Software; San Jose, CA, USA) under blind conditions.

## 5. Conclusions

In conclusion, we mimicked the essential features associated with the patient-reported rebound effect and identified in the altered ECoG power a possible major role of delta oscillations in promoting the seizures after LEV withdrawal. Then, we demonstrated that various neurosteroids were affected by the pharmacological treatment in chronic epileptic rats. A further investigation is needed to define the mechanism underlying the altered levels of neurosteroids, and the involvement of the hippocampal allopregnanolone in the development of SRSs during the treatment with LEV, and after its withdrawal.

## Figures and Tables

**Table 1:** Tissue levels of various neurosteroids in the hippocampus and neocortex of epileptic rats. Results are presented as mean ± standard error of the mean (SEM). Values under the limit of quantification (LOQ, Section 4.5) and outliers, identified by Grubbs’ test, were excluded.

Analytes (ng/mg)	Rats Treated with Saline (Mean ± SEM)	Rats Treated with LEV (Mean ± SEM)	*p*-Value of *t*-Test
Pregnenolone sulfate	0.01040 ± 0.00297	0.00042 ± 0.00018	*p* = 0.001
(hippocampus)	(*n* = 8)	(*n* = 11)	
Pregnenolone sulfate	0.00032 ± 0.00006	0.00014 ± 0.00003	*p* = 0.009
(neocortex)	(*n* = 8)	(*n* = 13)	
Pregnenolone	0.01070 ± 0.00215	0.00801 ± 0.00154	*p* = 0.312
(hippocampus)	(*n* = 8)	(*n* = 11)	
Pregnenolone	0.01080 ± 0.00041	0.00925 ± 0.00066	*p* = 0.106
(neocortex)	(*n* = 8)	(*n* = 13)	
Progesterone	0.00059 ± 0.00007	0.00068 ± 0.00005	*p* = 0.305
(hippocampus)	(*n* = 8)	(*n* = 10)	
Progesterone	0.00052 ± 0.00005	0.00045 ± 0.00008	*p* = 0.522
(neocortex)	(*n* = 8)	(*n* = 12)	
5α-Dihydroprogesterone	0.00106 ± 0.00032	0.00034 ± 0.00008	*p* = 0.018
(hippocampus)	(*n* = 7)	(*n* = 11)	
5α-Dihydroprogesterone	0.00020 ± 0.00003	0.00016 ± 0.00003	*p* = 0.490
(neocortex)	(*n* = 8)	(*n* = 12)	
Allopregnanolone	0.00030 ± 0.00006	0.00021 ± 0.00010	*p* = 0.530
(hippocampus)	(*n* = 7)	(*n* = 10)	
Allopregnanolone	0.00011 ± 0.00002	0.00036 ± 0.00009	*p* = 0.034
(neocortex)	(*n* = 8)	(*n* = 12)	
Pregnanolone	0.00015 ± 0.00005	0.00008 ± 0.00002	*p* = 0.120
(hippocampus)	(*n* = 3)	(*n* = 9)	
Pregnanolone	0.00667 ± 0.00146	0.00007 ± 0.00002	*p* ≤ 0.001
(neocortex)	(*n* = 8)	(*n* = 11)	

## Data Availability

Data available on request because of the agreement signed with granting agency.
